# Identification of Molecular Subtypes and a Prognostic Signature Based on Inflammation-Related Genes in Colon Adenocarcinoma

**DOI:** 10.3389/fimmu.2021.769685

**Published:** 2021-12-23

**Authors:** Chenjie Qiu, Wenxiang Shi, Huili Wu, Shenshan Zou, Jianchao Li, Dong Wang, Guangli Liu, Zhenbiao Song, Xintao Xu, Jiandong Hu, Hui Geng

**Affiliations:** ^1^ Department of General Surgery, Changzhou Hospital of Traditional Chinese Medicine, Changzhou, China; ^2^ Department of Pediatric Cardiology, Xinhua Hospital, Affiliated to Shanghai Jiao Tong University School of Medicine, Shanghai, China; ^3^ Department of Endodontics, Department of Oral & Maxillofacial Imaging, The Affiliated Stomatological Hospital of Nanjing Medical University, Nanjing, China

**Keywords:** colon adenocarcinoma, inflammation, immune infiltration, signature, molecule subtypes

## Abstract

Both tumour-infiltrating immune cells and inflammation-related genes that can mediate immune infiltration contribute to the initiation and prognosis of patients with colon cancer. In this study, we developed a method to predict the survival outcomes among colon cancer patients and direct immunotherapy and chemotherapy. We obtained patient data from The Cancer Genome Atlas (TCGA) and captured inflammation-related genes from the GeneCards database. The package “ConsensusClusterPlus” was used to generate molecular subtypes based on inflammation-related genes obtained by differential expression analysis and univariate Cox analysis. A prognostic signature including four genes (PLCG2, TIMP1, BDNF and IL13) was also constructed and was an independent prognostic factor. Cluster 2 and higher risk scores meant worse overall survival and higher expression of human leukocyte antigen and immune checkpoints. Immune cell infiltration calculated by the estimate, CIBERSORT, TIMER, ssGSEA algorithms, tumour immune dysfunction and exclusion (TIDE), and tumour stemness indices (TSIs) were also compared on the basis of inflammation-related molecular subtypes and the risk signature. In addition, analyses of stratification, somatic mutation, nomogram construction, chemotherapeutic response prediction and small-molecule drug prediction were performed based on the risk signature. We finally used qRT–PCR to detect the expression levels of four genes in colon cancer cell lines and obtained results consistent with the prediction. Our findings demonstrated a four-gene prognostic signature that could be useful for prognostication in colon cancer patients and designing personalized treatments, which could provide new versions of personalized management for these patients.

## Introduction

Colon cancer is one of the world’s most common diseases, with both high incidence and mortality rate. More than 1.9 million new colorectal cancer (including anus) cases and 935,000 deaths are expected in 2020, accounting for roughly one out of ten cancer cases and deaths (1). Overall, colorectal cancer (CRC) is the third most common cancer in the United States and the second leading cause of death. In many countries, the incidence of early-onset CRC (age at diagnosis <50 years) has increased, with the incidence increasing by 1% to 4% every year (2). For cancer categorization, prognosis prediction and therapeutic decision, the tumour, lymph node, metastasis (TNM) staging system, histological differentiation degree, and tumour sidedness have all been frequently employed. The main treatment for colon cancer is surgical excision of the primary tumour combined with adjuvant chemotherapy. The TNM staging system, however, is insufficient in actual practice for predicting prognosis and making treatment options for colon cancer patients. In the highly individualized precision medicine period, there is mounting evidence that genetic biomarkers have become essential (3, 4). It’s vital to seek biomarkers that can help forecast the likelihood of recurrence and death, allowing for early management and reducing the growing worldwide burden of CRC.

The tumour microenvironment (TME) plays an important role in the formation of tumours. The TME contains a variety of cell types, including infiltrating immune cells, cancer-associated fibroblasts, vascular cells, etc. These cell types have a reciprocal relationship and can govern tumour cell proliferation, cell death, growth suppressor evasion, energy metabolism, and immune evasion, as well as angiogenesis and tumour cell invasion, in a cell non-autonomous manner (5). The TME contains various soluble substances, including cytokines, chemokines, inflammatory factors and cellular metabolic products, in addition to the physical components within the tumour. All phases of tumourigenesis are influenced by inflammation inside the TME, and inflammation can drive the plasticity of both tumour cells and surrounding cells within the TME to a great extent (6). The inflammatory environment affects the development of many colorectal tumours (either preceding tumourigenesis, tumour elicited or therapy induced) (5). Therefore, tumour progression and patient survival reflect the complex cellular and molecular interactions between tumour and host immune system (7). At present, there is no reliable model according to inflammation-related genes to predict the patients’ prognosis in colon cancer.

In our study, we developed a prognostic signature including four inflammation‐related genes (IRGs) with differential expression analysis and univariate and multivariate Cox regression to evaluate prognosis independently in colon adenocarcinoma (COAD) from The Cancer Genome Atlas (TCGA) database and validated the performance of the signature from the Gene Expression Omnibus (GEO) database. We also analysed the survival stratification, somatic mutations, nomogram construction, chemotherapy response prediction and small molecule drug prediction based on risk characteristics.

## Materials and Methods

### Data Source

RNA-seq data, relevant clinical information, simple nucleotide variation data were downloaded from TCGA database. GSE17538 was downloaded from GEO database to validate the signature. The list of inflammation-related genes was obtained from the GeneCards database.

### Analysis of Differentially-Expressed Inflammation-Related Genes (DE-IRGs)

DE-IRGs were obtained by comparing 41 normal and 480 COAD tissues in TCGA and the filter criteria was |log Foldchange (FC) | > 1 and false discovery rate (FDR) < 0.05. DE-IRGs were then utilized to perform Gene Ontology (GO) and Kyoto Encyclopedia of Genes and Genomes (KEGG) analysis. Protein-protein interaction (PPI) was conducted by the STRING database and Cytoscape. Hub genes and modules were analyzed by “cytohubba” and “MCODE” plugins in Cytoscape.

### Cluster Analysis

Univariate Cox regression analysis was used to screened out prognosis-related DE-IRGs. Package “ConsensusClusterPlus” was used to perform cluster analysis to identify inflammation-related molecules subtypes. We performed Kaplan-Meier (K-M) analysis to compare the prognosis between the two clusters. The heatmap was used to display the correlation between clusters and clinical parameters, which analyzed by chi-square test.

### Construction and Validation of the Prognostic Signature

Multivariate Cox regression analysis was further used to search for independent genes for COAD to construct the prognostic signature. The coefficient of the selected genes was displayed by Graphpad software. K-M analysis and Receiver Operation Characteristic (ROC) curve were used to judge the prognostic value of the signature. GSE17538 was used to validated the prognostic signature. Univariate and multivariate Cox analysis were used to identify whether the signature was an independent risk factor. Based on the clinicopathology parameters, we performed correlation analysis between risk scores and clinical features, stratification analysis and nomogram construction. Calibration plots were used to compare the consistency between predicted probabilities of 3.5-, 5- and 7.5-year survival and actual ones.

### Gene Set Enrichment Analysis (GSEA) Based on the Signature

We conducted GSEA to analyze the pathways enriched in the high-risk group to explore the potential mechanisms. Reference gene sets included hallmark, c2kegg, c2biocartar and c5go. The screening conditions were |normalized enrichment score (NES)| > 1, nominal (NOM) p-value < 0.05 and FDR q-value < 0.25.

### Immune Landscape Analysis

Four immune-related algorithms were used to analyze the immune landscape between the high- and low-risk groups. The activity of immune cell or immune function, immune pathway of each sample was calculated by single sample GSEA (ssGSEA). The marker genes of different immune cells were obtained from previous studies (8) and listed in **Table S1**. ESTIMATE algorithm was used to calculate the immune score, stromal score, estimate score and tumour purity according to the proportion of immune cells and stromal cells. Prediction of the composition of infiltrating immune cells in each tumour sample was derived from the TIMER database and CIBERSORT algorithm. We also compared the expression of MHC molecules based on the cluster analysis and the signature.

In the aspect of immune checkpoint, five common immunoinhibitors (PD-L1, CTLA4, HAVCR2, LAG3 and PD1) were firstly compared according to clusters and risks. It was known that higher Tumour Immune Dysfunction and Exclusion (TIDE) score was associated with poorer immune checkpoint blocking treatment and shorter survival. TIDE score will help doctors select patients who are more suitable for immune checkpoint therapy. Thus, we calculated the TIDE score of COAD patients in TCGA through the TIDE database.

### Tumour-Related Scores and Tumour Stemness Indices (TSIs) Analysis

Previous studies have found that patients with poor prognosis for gliomas have higher angiogenic activity score, mesenchymal-Epithelial-mesenchymal-transition (EMT) score, tumourigenic cytokines score and stemness score. Relevant marker genes were listed in **Table S2**. We applied ssGSEA algorithm to calculate the scores of angiogenic activity, mesenchymal-EMT, tumourigenic cytokines and stemness of each tumour samples. TSIs were associated with active biological processes in stem cells and a higher degree of tumour dedifferentiation. We obtained TSIs of TCGA patients from a previous study (9).

### Gene Mutation Analysis

Based on the somatic mutation data from TCGA, we conducted gene mutation through “maftools” package. We then calculated tumour mutation burden (TMB) of each patient and compared TMB between the high- and low-risk groups. Survival analysis was also performed according to TMB score. At the same time, somatic mutations of the selected genes in the signature were displayed through the cBioPortal database.

### Chemotherapy Response and Small-Molecule Drugs

We predicted chemotherapy response of the chemotherapy drugs for COAD patients through the Genomics of Drug Sensitivity in Cancer (GDSC) database. The half maximal inhibitory concentration (IC50) was calculated by package “pRRophetic” and used to evaluate the response of patients to chemotherapy drugs. The connectivity map (cMap) database is a bio-application database combining small-molecule drugs, gene expression and disease. Based on the up-regulated and down-regulated genes compared between the low- and high-risk groups, we obtained predicting drugs that might induce or reverse tumour biological process. P-value < 0.05 and the enrichment score ranged from -1 to 0 indicated that the potential drug may be the potential new target candidates for COAD patients. The 3D structure figures of these candidate drugs were obtained from the PubChem database.

### Real-Time Polymerase Chain Reaction (RT-PCR)

Total RNA was extracted using TRIzol reagent (Invitrogen, Thermo Fisher Scientific, Inc.) from six colon cancer cells (HCT116, SW480, HT29, LOVO, RKO, DLD-1) and a normal epithelial colon cell NCM460. The PrimeScript™ RT reagent kit (TaKaRa) was used for reverse transcriptase reaction. The mRNA expression level of TIMP1, PLCG2, BDNF and IL13 was normalized by GAPDH. The primers of the five genes were listed in **Table S3**. Fold differences were calculated for each group using normalized CT values.

## Results

### Identification of Differential Inflammation-Related Genes and Biological Function Analysis

**Figure 1:**
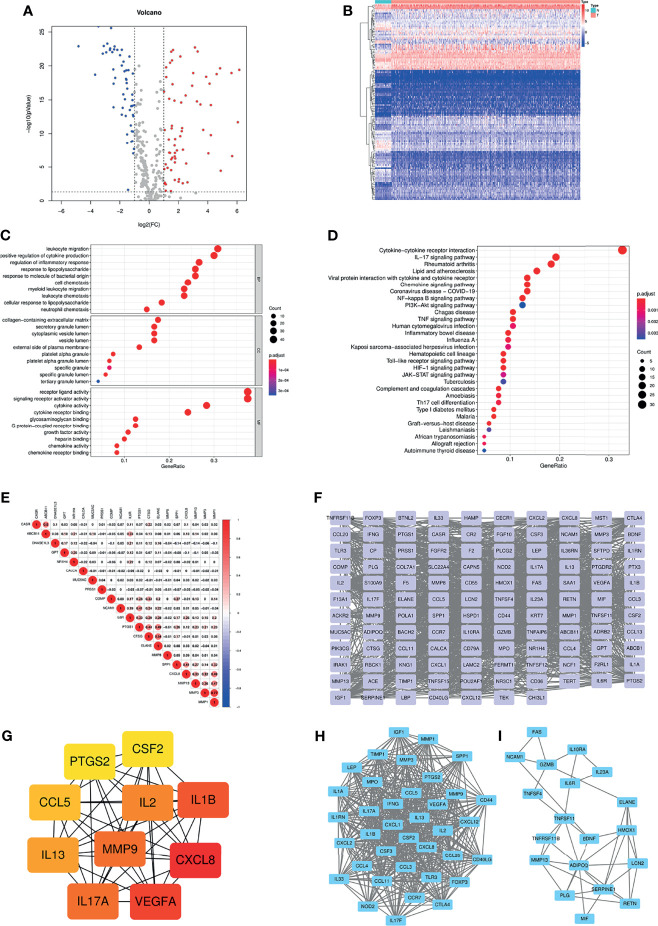
**(A)** Volcano plot of 66 up-regulated and 54 down-regulated IRGs in COAD (FDR < 0.05 and |logFC| > 1). **(B)** Heatmap of 120 DE-IRGs between normal colon and COAD tissues. **(C)** The top ten enriched terms in GO analysis belonged to BP, CC, and MF for DE-IRGs. **(D)** The top thirty enriched terms in KEGG analysis. **(E)** The correlations between the top ten up-regulated and down-regulated IRGs. **(F)** PPI network of the DE-IRGs according to the STRING database. **(G)** The hub genes obtained from “cytohubba” plugin. **(H, I)** The two modules obtained from “MCODE” plugin. IRGs, Inflammation-related genes; COAD, Colon adenocarcinoma; FDR, False discovery rate; FC, Fold change; DE-IRGs, Differentially-expressed IRGs; GO, Gene Ontology; BP, Biological process; CC, Cell component; MF, Molecular function; KEGG, Kyoto Encyclopedia of Genes and Genomes; PPI, Protein-protein interaction.

Based on the GeneCards database, 357 inflammation-related genes were obtained, and the screening criteria were protein coding genes and relevance scores greater than 5. The main flow of this study is shown in **Supplementary Figure S1**. Subsequently, by differential analysis of COAD and normal colon tissues, 66 upregulated genes and 54 downregulated genes were obtained (**Figures 1A, B**). GO and KEGG pathway enrichment analysis showed that the above differentially expressed genes were mainly enriched in immune, inflammatory, cytokine-cytokine receptor and tumour-related biological functions and signalling pathways (**Figures 1C, D**). In addition, correlations between the top 10 upregulated and downregulated genes were also displayed (**Figure 1E**). The PPI network was explored using the STRING database and visualized using Cytoscape (**Figure 1F**). The top ten hub genes were obtained by ranking with degree (**Figure 1G**), while two modules were identified based on MCODE (**Figures 1H, I**).

### Identification of Inflammation-Associated Clusters and Correlation Analysis Between Clusters and the Tumour Immune Microenvironment, Tumourigenesis Scores, and TSIs

**Figure 2:**
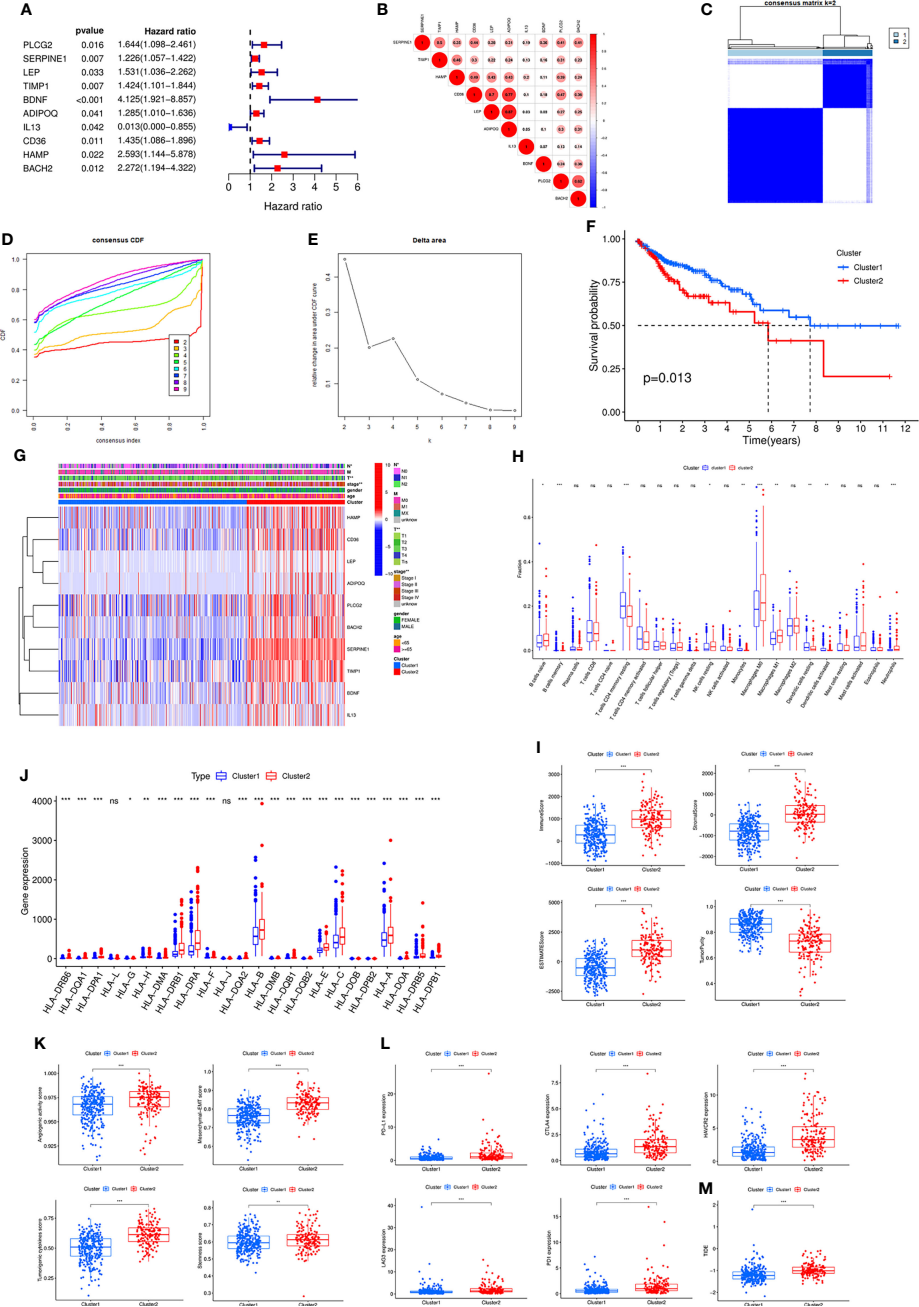
**(A)** Forest plot of ten prognostic-related DE-IRGs through univariate Cox analysis. **(B)** The correlations between the ten genes. **(C)** Consensus clustering matrix when k = 2. **(D)** Consensus clustering CDF with k valued 2 to 9. **(E)** Relative change in area under CDF curve for k = 2. **(F)** KM curve of the survival difference between cluster 1 and cluster 2. **(G)** Heatmap of the ten genes between the two clusters and the correlations of the clusters and clinical parameters. Immune cell infiltration using CIBERSORT **(H)**, immune and stromal scores using ESTIMATE **(I)**, the expression of MHC molecules **(J)**, angiogenic activity, mesenchymal-EMT, tumourigenic cytokines and stemness scores **(K)**, five common immunoinhibitors **(L)**, and TIDE score **(M)** between the two clusters. CDF, Cumulative distribution function; KM, Kaplan–Meier; EMT, Epithelial-mesenchymal-transition; TIDE, Tumour Immune Dysfunction and Exclusion.

By univariate Cox analysis, we obtained 10 genes associated with prognosis, in which IL13 was a protective factor and the other 9 genes were risk factors (**Figure 2A**). Correlation analysis showed that most genes were correlated with each other (**Figure 2B**). The ten prognosis-related genes were further utilized for cluster analysis. The cluster effect was best when COAD patients were clustered into two subgroups, and the subgroup internal consistency and stability were good (**Figures 2C–E**). Survival analysis showed a better prognosis for Cluster 1 than Cluster 2 (**Figure 2F**). The heatmap revealed the gene expression differences between the 2 clusters and the significant correlation with clinicopathological parameters, such as stage, T stage and N stage (**Figure 2G**).

Subsequently, various algorithms were used to analyse the differences in immune infiltration between the two clusters. We found significant differences in immune cells and immune-related functions or pathways between the two clusters (**Supplementary Figure S2A**). The TIMER algorithm showed that Cluster 2 was associated with more immune cell infiltrates, such as CD4+ T cells, CD8+ T cells, neutrophils, macrophages and dendritic cells (**Supplementary Figure S2B**). The CIBERSORT algorithm revealed similar results (**Figure 2H**). The ESITIMATE algorithm showed that Cluster 2 had a higher immune score, stromal score, ESITIMATE score and lower tumour purity (**Figure 2I**). We also found that Cluster 2 was related to higher expression of many MHC molecules (**Figure 2J**).

In addition, angiogenic activity, mesenchymal EMT, tumourigenic cytokines and stemness scores were significantly higher in Cluster 2 (**Figure 2K**). Gui et al. found that the hypoxia-immune risk score was negatively associated with EREG-mRNAsi and ENHsi. Higher EREG-mRNAsi and ENHsi levels meant better prognosis (10). In this study, Cluster 2 was found to be relevant to lower TSIs (**Supplementary Figure S2C**).

Because the two clusters differed significantly regarding immune infiltration, we evaluated the correlation with the five common immune checkpoints. Cluster 2 had higher expression of PD-L1, CTLA4, HAVCR2, LAG3 and PD1 (**Figure 2L**). Cluster 2 was associated with a higher TIDE score (**Figure 2M**).

### Construction of an Inflammation-Related Signature and a Nomogram Based on the Signature

**Figure 3:**
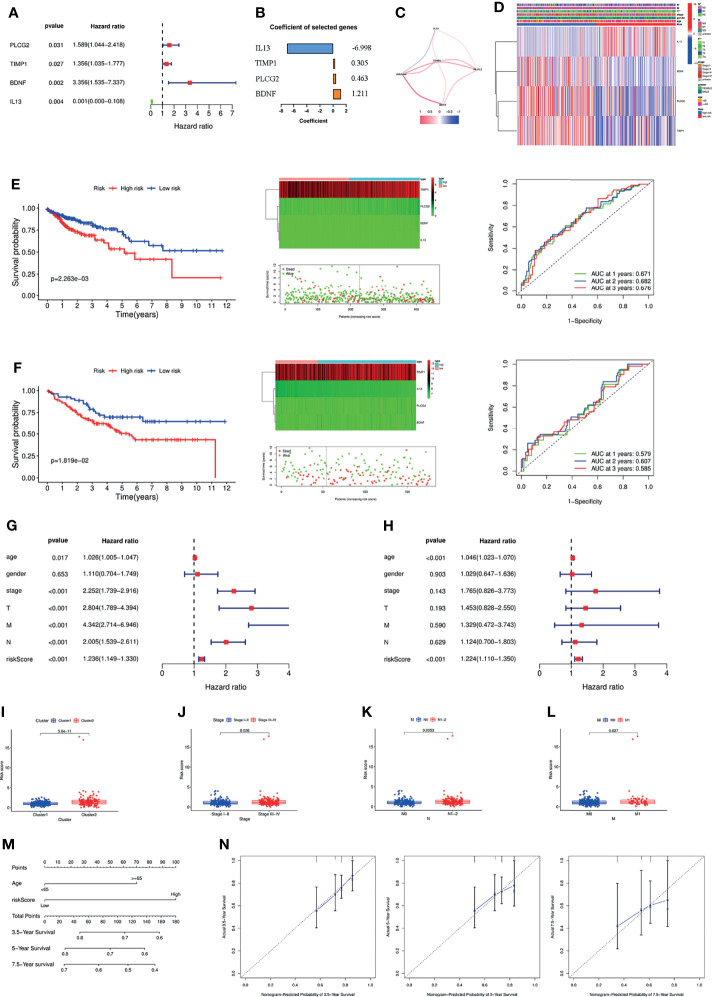
**(A)** Forest plot of the four genes selected in the signature through multivariate Cox analysis. **(B)** Coefficients of the four genes included in the signature. **(C)** The correlations between the signature and the four genes. **(D)** Heatmap of the association between the expression levels of the four genes and clinicopathological features. Survival analysis, heatmap, survival status accompanied with the risk score and ROC analysis in TCGA cohort **(E)** and GSE17538 cohort **(F)**. The signature was an independent risk factor for COAD patients in TCGA cohort according to univariate **(G)** and multivariate Cox analysis **(H)**. The differences of the risk score between different groups according to clinicopathological features, e.g., clusters **(I)**, tumour stage **(J)**, lymph node status **(K)**, and metastasis **(L)**. **(M)** Nomogram based on risk score and age. **(N)** Calibration plots of the nomogram for predicting the probability of 3.5-, 5- and 7.5-year survival. ROC, Receiver operating characteristic; TCGA, The Cancer Genome Atlas.

To further screen the genes included in the model, multifactor Cox regression was performed, and four genes were selected into the signature (**Figure 3A**). The coefficient of each gene in the signature is shown in **Figure 3B**. **Figure 3C** shows the association of the risk score with PLCG2, TIMP1, BDNF and IL13. The risk score was associated with T stage and N stage (**Figure 3D**). Patients with a high-risk score had a poorer prognosis than those with a low-risk score, and the AUC of the signature was 0.671 at one year (**Figure 3E**). The GSE17538 dataset was used to validate the signature, which had good efficiency (**Figure 3F**). General characteristics of patients in TCGA and GSE17538 were shown in **Table S4**. Stratified analysis showed that the signature could significantly differentiate patient prognosis in almost all clinical subgroups, i.e., patients in the high-risk group had a poorer prognosis (**Supplementary Figure S3**). In addition, the signature was an independent risk factor by univariate and multivariate Cox regression analysis (**Figures 3G, H**). We finally analysed the differences in risk score between subgroups based on different clinicopathological parameters. The results showed that Cluster 2, stage III-IV, N1–2 and M1 patients had higher risk scores, meaning that the higher the risk score was, the more advanced the tumour (**Figures 3I–L**).

Based on the results of the above multivariate Cox regression analysis, we included age and the signature in the construction of a nomogram, and the signature was the most important factor in the nomogram (**Figure 3M**). Calibration plots showed that the actual 3.5-, 5- and 7.5-year survival times were highly consistent with the predicted survival times (**Figure 3N**).

### Estimation of Tumour Immune Cell Infiltration and Immune Checkpoint Inhibitors According to the Signature

**Figure 4:**
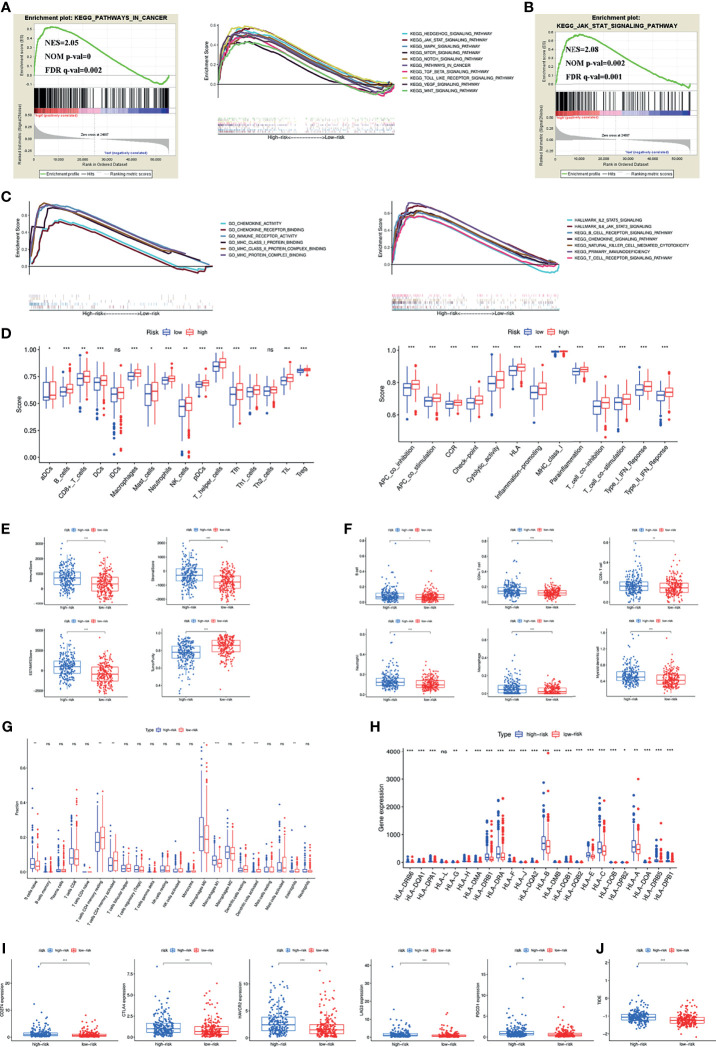
**(A)** Pathways related to tumour development and progression enriched in the high-risk group. **(B)** JAK-STAT signaling pathway was the most relevant KEGG pathway in the high-risk group. **(C)** Multiply pathways associated with immune, chemokine and MHC molecules enriched in the high-risk group. Immune cell infiltration and immune-related functions or pathways **(D)**, immune and stromal scores **(E)**, immune cell infiltration using TIMER **(F)** and CIBERSORT **(G)**, MHC molecules expression level **(H)**, five common immunoinhibitors **(I)** and TIDE score **(J)** between the high- and low-risk groups. (*P < 0.05; **P < 0.01; ***P < 0.001; ns, not significant).

To investigate the possible involvement of pathways regulating tumourigenesis in the high-risk group, GSEA was performed. The results showed that multiple classic tumour-related pathways were enriched in the high-risk group, and pathways in cancer (NES = 2.05, NOM p value = 0, FDR q-value = 0.002) were significantly enriched (**Figure 4A**). The JAK-STAT signalling pathway was the most relevant KEGG pathway, with NES = 2.08, NOM p value = 0.002 and FDR q-value = 0.001 (**Figure 4B**).

Previous findings suggested that both the inflammatory response and the tumour microenvironment played an important role in tumour development (11, 12). We first found that many immune-related pathways were associated with the high-risk group by GSEA (**Figure 4C**). Therefore, the relationship between the signature and tumour immune microenvironment was further investigated. Through the ssGSEA algorithm, the high-risk group had higher immune cell infiltration and more immune-related functions or pathways than the low-risk group (**Figure 4D**). The ESTIMATE algorithm checked the above results and found higher immune scores, stromal scores, ESTIMATE estimation scores and lower tumour purity in the high-risk group (**Figure 4E**). Immune cells that predominantly infiltrated in the high-risk group included B cells, CD4+ T cells, CD8+ T cells, neutrophils, macrophages and dendritic cells (**Figure 4F**). Eosinophils were also higher in the high-risk group (**Figure 4G**). Additionally, we detected the expression of MHC molecules and found that they were significantly increased in the high-risk group (**Figure 4H**).

Five immune checkpoint inhibitors (PD-L1, CTLA4, HAVCR2, LAG3 and PD1) were highly expressed in the high-risk group (**Figure 4I**). The high-risk group was also associated with a higher TIDE score (**Figure 4J**).

### Correlation of Angiogenic Activity, Mesenchymal EMT, Tumourigenic Cytokines, Stemness Scores and TSIs With the Signature

**Figure 5:**
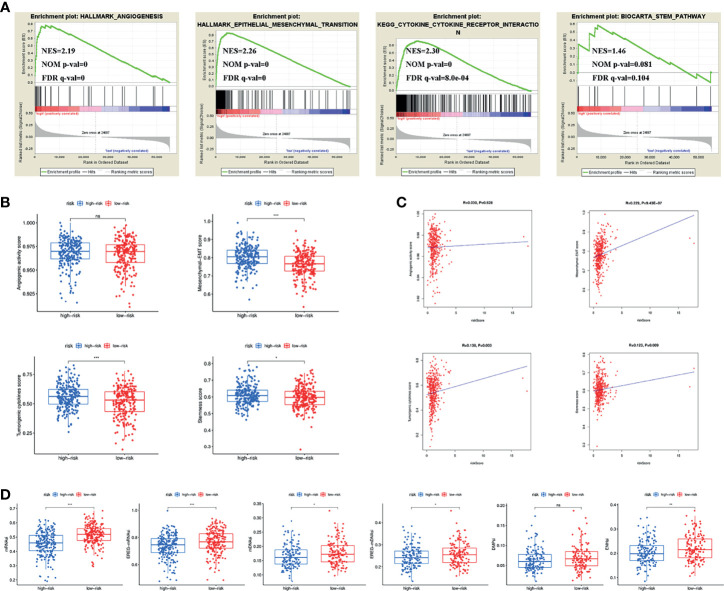
**(A)** Pathways related to angiogenesis, EMT, cytokine-cytokine receptor interaction and stemness enriched in the high-risk group. **(B)** Differences of angiogenic activity, mesenchymal-EMT, tumourigenic cytokines and stemness scores between the high- and low-risk groups. **(C)** The correlation of the risk score and angiogenic activity, mesenchymal-EMT, tumourigenic cytokines and stemness scores. **(D)** Differences of TSIs between the two groups. TSIs, Tumour stemness indices. (*P < 0.05; **P < 0.01; ***P < 0.001; ns, not significant).

Previous results found that different clusters were associated with angiogenic activity, mesenchymal EMT, tumourigenic cytokines and stemness scores. We therefore wanted to explore whether these four tumour-related functions were involved in the underlying mechanisms of the signature. GSEA results showed that angiogenesis, epithelial-mesenchymal transition, cytokine-cytokine receptor interaction and the stem pathway were enriched in the high-risk group (**Figure 5A**). We subsequently calculated angiogenic activity, mesenchymal EMT, tumourigenic cytokines and stemness scores for COAD patients. **Figure 5B** shows that the high-risk group had higher mesenchymal EMT, tumourigenic cytokines and stemness scores. **Figure 5C** shows the correlation of the risk score with four indices, suggesting that the risk score was positively associated with the mesenchymal EMT score (R=0.229, p=9.43e-07), tumourigenic cytokine score (R=0.138, p=0.003) and stemness score (R=0.123, p=0.009). In addition, the high-risk group had lower TSIs, such as mRNAsi, EREG-mRNAsi, mDNAsi, EREG-mDNAsi and ENHsi (**Figure 5D**).

### Comparison of Somatic Mutation and TMB in the Signature

**Figure 6:**
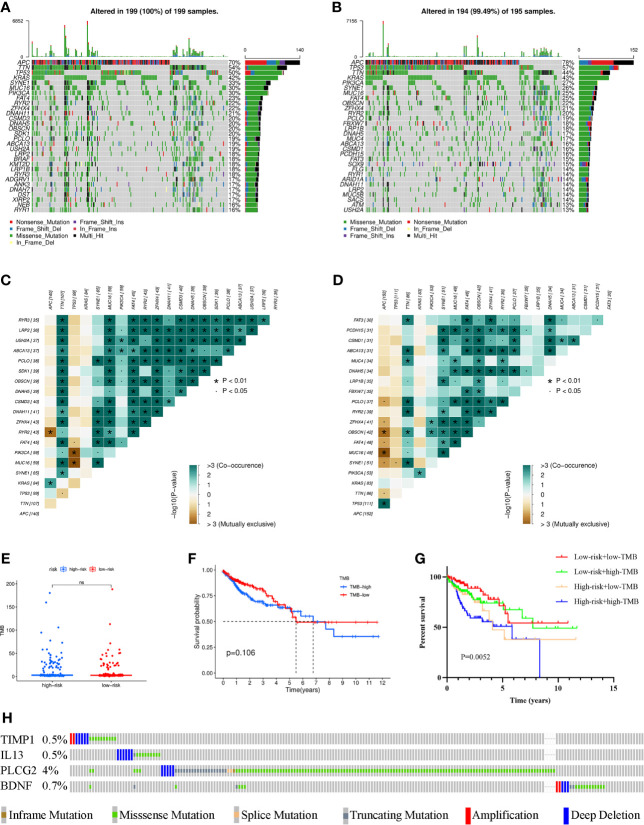
Waterfall maps of the somatic mutations in the high-risk group **(A)** and the low-risk group **(B)**. Heatmap of co-occurrence and mutually exclusive mutations of the differently mutated genes in the high-risk group **(C)** and the low-risk group **(D)**. *p < 0.01. **(E)** Comparison of TMB between the high- and low-risk groups. **(F)** Difference in overall survival between high TMB and low TMB groups. **(G)** Difference in overall survival based on TMB and risk score. **(H)** Mutation rates of four genes (TIMP1, IL13, PLCG2, BDNF) in COAD patients from the cBioPortal database. (ns, not significant).

To investigate the differences in genomic mutations between the high- and low-risk groups, we downloaded simple nucleotide variation data from TCGA. APC (70%), TTN (54%), TP53 (50%), KRAS (42%) and SYNE1 (33%) were the top 5 genes with the highest mutation frequencies in the high-risk group, while APC (78%), TP53 (57%), TTN (44%), KRAS (43%) and PIK3CA (27%) were the top 5 genes in the low-risk group (**Figures 6A, B**). In addition, somatic mutation interactions were detected. Gene mutation cooccurrence existed between most genes, and mutually exclusive APC-RYR2 mutations were discovered in the high-risk group (**Figure 6C**). Gene mutation cooccurrence phenomena also occurred frequently in the low-risk group (**Figure 6D**). TMB was also compared between the two groups, and no significant difference was found (**Figure 6E**). There was no difference in survival time between the high-TMB and low-TMB groups (**Figure 6F**). After combining with our model, the prognosis of the high-risk + high TMB group was significantly worse than that of the low-risk + low TMB group (**Figure 6G**). Finally, we detected the mutation rates of the four genes in the signature and found that the mutation rates were all low (**Figure 6H**).

### Prediction of the Chemotherapy Response and Screening of Small-Molecule Drugs

**Figure 7:**
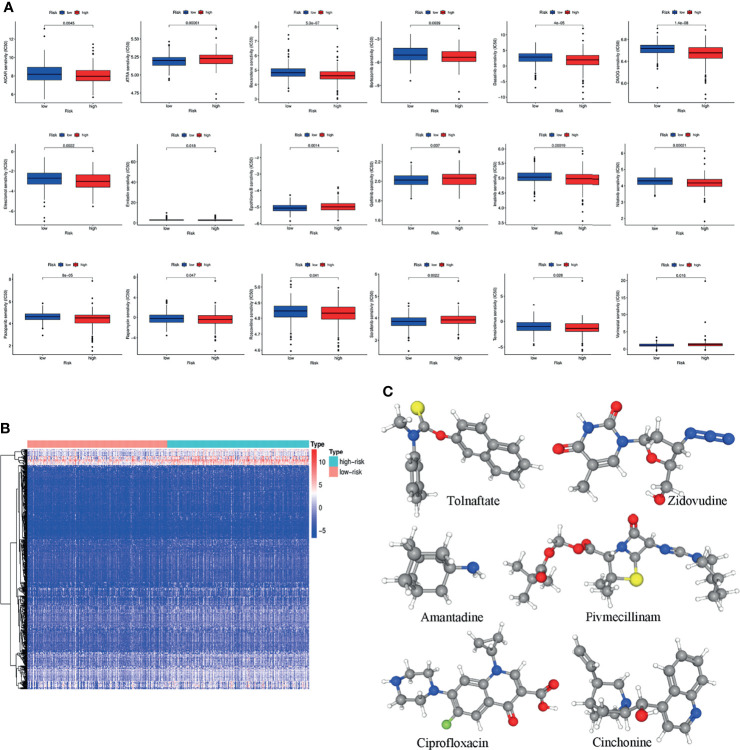
**(A)** The differences in the chemotherapy response of common chemotherapy drugs between the high- and low-risk groups. **(B)** Differentially expressed genes between the high- and low-risk groups. **(C)** The 3D structure of six potential target drugs screened out from the cMap database. (*P < 0.05; **P < 0.01; ***P < 0.001; NS, not significant).

GDSC was used to predict the chemotherapy response of the common chemotherapy agents between the two groups (**Figure 7A**). The sensitivity of many chemotherapeutic agents differed significantly between the high- and low-risk groups (p<0.001 for dasatinib, p=0.0022 for elesclomol, p=0.0014 for epothilone B, p=0.037 for gefitinib, p<0.001 for imatinib, p<0.001 for nilotinib, p<0.001 for pazopanib, p=0.0022 for sorafenib, p=0.028 for temsirolimus).

In addition, the cMap database was used to screen small-molecule drugs for COAD. By comparing the low- and high-risk groups, 501 upregulated and 12 downregulated genes were obtained (**Figure 7B**). The six most associated small-molecule drugs were screened as potential target drug candidates for COAD patients based on the differentially expressed genes. The 3D structures of tolnaftate, amantadine, ciprofloxacin, zidovudine, pivmecillinam and cinchonine were displayed through the PubChem database (**Figure 7C**).

### The Expression of PLCG2, TIMP1, BDNF and IL13 in Different Colon Cancer Cell Lines

**Figure 8:**
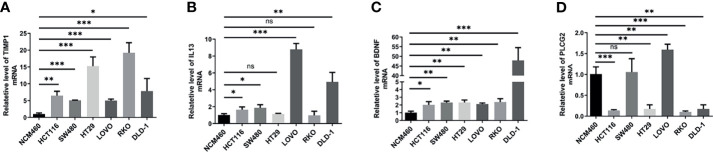
The mRNA expression levels of TIMP1 **(A)**, IL13 **(B)**, BDNF **(C)** and PLCG2 **(D)** in different cell lines (NCM460, HCT116, SW480, HT29, LOVO, RKO, DLD-1) were measured by qRT-PCR. Results were normalized to reference gene GAPDH. Data are shown as the mean ± SEM, two-tailed unpaired t test was used for statistical calculation for each marker, n=3 independent experiments. (*P < 0.05, **P < 0.01, ***P < 0.001; ns, not significant).

To verify the results of our data analysis, we extracted total RNA from different tumour cell lines (HCT116, SW480, HT29, LOVO, RKO, DLD-1) and the normal epithelial colon cell line NCM460 and measured the mRNA expression levels of TIMP1, PLCG2, BDNF and IL13. qRT–PCR assays showed that the mRNA expression levels of TIMP1 and BDNF were significantly higher in colon cancer cells than in NCM460 cells (**Figures 8A, B**). Excluding HT29 and RKO, the mRNA levels of IL13 in HCT116, SW480, LOVO, and DLD-1 cells were significantly increased (**Figure 8C**). According to the database analysis, PLCG2 had low expression in tumour tissues, and the results of the experiments confirmed this conclusion (**Figure 8D**).

## Discussion

In this study we revealed an inflammation-related signature made up of four genes (PLCG2, TIMP1, BDNF, and IL13) that can predict clinical outcomes and treatment responses in COAD patients in this investigation. Our findings could increase the accuracy of survival probability predictions for COAD patients.

In recent years, there has been an increase in the number of studies on the effects of inflammation on the occurrence, development, and prognosis of colon cancer. The main risk factors for colon cancer are chronic inflammation caused by infection, abnormal immune response, or environmental factors, as well as chronic inflammation of the gastrointestinal tract caused by poor eating habits (13, 14). Chronic inflammation can initiate and promote tumourigenesis in inflammation-related tumourigenesis by inducing DNA damage or silencing tumour suppressor genes (15). Colon cancer can also trigger inflammation. During the progression of colon cancer, oncogene activation and loss of tumour suppressor genes further lead to normal cell death and disruption of the intestinal epithelial barrier, allowing microbial products from the lumen to induce the production of inflammatory factors, growth factors and chemokines in the tissues (16). Recruitment of inflammatory immune cells to the tumour site promotes tumour growth and distant metastases. Therapy-induced inflammation is a major correlate of therapeutic response and relapse, and it can have a significant impact on the course of colon cancer (17). TME cells may release growth factors and cytokines such as WNT, epidermal growth factor, TNF, IL-17, and IL-6 in response to therapy-induced tumour cell death, which promotes the survival of residual tumour cells and leads to treatment resistance (6). Based on large-scale sequencing data, we are now well equipped to construct a prognostic signature that can be used to guide personalized treatment and predict adverse treatment effects (18).

IL-13 is an immunomodulatory factor that can regulate inflammation and immune response (19). IL-13 is produced by a variety of immune cells, such as CD4-T cells, basophils, eosinophils and NK cells, which are essential for the induction and persistence of type 2 immune responses. The expression levels of IL-13 were higher in COAD tissues than in adjacent normal tissues. Higher IL-13 expression was related with a longer survival time in an immunohistochemical study with 359 CRC samples (20). Furthermore, a research involving 241 CRC patients found that serum IL-13 levels were significantly lower in advanced cancer patients, and lower serum IL-13 levels were significantly associated with a poorer prognosis (21). Secretion of IL-13 by Innate Lymphoid Cells (ILC2s) is crucial for the migration of activated dendritic cells (DCs) to the draining lymph nodes (22), where T cell priming and activation takes place. Additionally, IL-13 can promote epithelial cells to secrete eosinophil chemokine eotaxin and recruit eosinophils. Activated eosinophils can secrete chemokines CXCL9, CXCL10, CCL17, and recruit CD4+T and CD8+T cells into the tumour microenvironment. Ultimately, the recruited T cells produce anti-tumour effects (23). During inflammation resolution, Regulatory T (Treg) cells secreted IL13 to promote macrophage efferocytosis and enhanced apoptotic cell internalization (24).

As an intrinsic inhibitor of matrix metalloproteinases (MMPs), the imbalance between tissue inhibitor of metalloproteinases 1 (TIMP1) and MMPs is an important factor leading to the development of gastrointestinal malignancies (25). The interaction between the C-terminal domain of TIMP1 protein and tetraspanin CD63 can induce the conformational activation of integrin b1 and activate the MAPK signal, thereby inducing tumourigenesis (26). TIMP1 can activate fibroblast-like hepatic stellate cells (HSCs) through TIMP-1/CD63 signal and secrete SDF-1 to attract neutrophils that promote metastasis. In this process, TIMP1 significantly increases the sensitivity of the liver to circulating tumour cells and creates a tumour microenvironment that promotes tumour liver metastasis (27, 28). Similarly, TIMP1 can also activate cancer-related fibroblasts Cells (CAF) promote the growth of primary tumours (29). This is why we can observe that TIMP1 elevation is associated with the poor prognosis of human tumours in many clinical trials (30). TIMP1 was found to be a sensitive biomarker in patients with metastatic colon cancer, and higher TIMP1 levels were linked to lymph node metastases, vascular invasion and distant metastasis in CRC patients (31, 32). Low expression of TIMP1 decreased the invasion and migration of SW480 and HCT116 colon cancer cells (33).

As previously reported, the brain-derived neurotrophic factor (BDNF) is substantially elevated in COAD compared with nontumour tissues according to a clinical research (34). Additionally, BDNF promoted the proliferation of human colon cancer cells, and its levels were significantly elevated in tumours with poor prognosis. BDNF/TrkB signaling participates in the formation of drug resistance in HT-29 colon cancer cells through an EGFR-dependent mechanism (35). Human BDNF enhanced the migratory activities of colon cancer cell lines SW480 and HCT116, through modulating VEGF/HO-1 activation *via* the ERK, p38, and PI3K/Akt signaling pathways (36). The enriched environment (EE) stimulates the hypothalamus to release BDNF, which enhances adaptive immunity and further affects the progression of cancer. The spleen, bone marrow and blood of mice living in the environment have a higher proportion of Natural killer cells (NK), and EE-stimulated tumour-bearing mice have observed increased maturity of NK cells (37). Overexpression of BDNF in the hypothalamus replicates the EE-induced NK cell phenotype, while the knock-out of BDNF in the hypothalamus abolishes EE-induced NK regulation (38). In addition, BDNF is also involved in EE-induced T cell regulation in primary and secondary lymphoid tissues, including the reduction of the total number of splenic T cells and the change (ratio Decrease) of CD4 T helper to CD8 cytotoxic T lymphocytes (CTL) in secondary lymphoid tissue (SLT) (39, 40). These results indicate that BDNF is an important immunomodulatory molecule that enhances the body’s response to EE and induces downstream immune changes.

PLCG2 is an enzyme that converts phosphatidylinositol 4,5-bisphosphate (PIP2) into diglycerides (DAG) and inositol triphosphate (IP3), and is required for function of many immune cells, including B cells, NK cells, mast cells, and macrophages (41–43). The ip3 catalyzed by PLCG2 and the subsequent calcium signal ensure that B lymphocytes have the correct developmental results and antigen-specific responses (44). A gain-of-function mutation of PLCG2 leads to hyperreactive external calcium entry in B cells and expansion of innate inflammatory cells. The mutant confirmed that PLCG2 is a key regulator in autoimmune and inflammatory diseases mediated by B cells (45). PLCG2 is essential for NK cell responses to malignant and virally infected cells. PLCG2 deficient NK cells failed to secrete cytotoxic granules and lost exocytosis of cytotoxic granules, due to defective calcium mobilization (46). Lipopolysaccharide (LPS) and peptidoglycan (PGN) stimulated the phosphorylation of PLCG2 to induce Ca2+ mobilization in macrophages and dendritic cells and enhanced the production of cytokines, meaning that the PLCG2 signalling pathway played a significant part in bacterial ligand-induced responses in cell-mediated immunity (47). PLCG2 is involved in the proliferation and migration of many cancers (48). Because of its overexpression following ionizing radiation, PLCG2 has been identified as a potential biomarker of radiation exposure (49). These findings indicated the considerable potential of PLCG2 as a prognostic indicator and drug target.

We believed that the prognostic signature we established was immunological in nature, and stratification based on immune phenotype was useful. We also simulated the role of drugs in different reactions, showing that the sensitivity of multiple drugs was substantially varied between the high-risk and low-risk groups. By comparing the differentially expressed genes between the two groups, we predicted six potential therapeutic drugs for COAD patients. The proliferation of colonic epithelial adenocarcinoma cell line was suppressed by ciprofloxacin in a concentration- and time-dependent manner (50). Additionally, ciprofloxacin might be more effective than metronidazole in the treatment of pouchitis following ileal pouch anal anastomosis (IPAA) surgery (51). The thymidine analogue zidovudine is currently used to treat HIV-infected patients. Intraperitoneal injection of zidovudine combined with methotrexate or fluorouracil in mice bearing human colon cancer xenografts significantly inhibited tumour growth (52, 53). The same results were obtained in the human colon cancer cell lines HCT-8, SW620, SW480, and COLO-320DM in a preclinical analysis (54). Cinchonine increased doxorubicin absorption in cancer cells (DHD/K12/PROb rat colon cells) and was effective in lowering tumour mass and improving survival in rats injected intraperitoneally with deoxydoxorubicin (55). There is currently no research that can clarify the role of other drugs (tolnaftate, amantadine, pivmecillinam) in the development of colon cancer. In the follow-up research process, we will attempt to explore the relationship between these drugs and colon cancer.

Our study also had some limitations. First, we only validated the signature using retrospective data from the GEO database, and we should explore its clinical value through more prospective studies in the future. Second, further *in vivo* and *in vitro* investigations are required to examine the role of the four selected genes in the development of colon cancer. Third, this study only analyzed the correlation between risk model and immune cells, immune function, MHC molecules, immune checkpoints and immunotherapy and only indicated that there was a possible relationship between the risk model and immune status. In the future, we will continue to collect enough samples to evaluate the value of this model in combination with immunotherapy, and evaluate whether there is a difference in the benefit of immunotherapy between the high- and low-risk groups. Fourth, the broad impact of the signature on multiple drug sensitivity warrants our focus on potential drug screening.

## Conclusions

In summary, this study identified molecular subtypes based on IRGs in colon cancer and used IRGs to construct a prognostic signature. The immune landscape, gene mutation status and drug sensitivity between different molecular subtypes and between different risk groups were also analysed. The signature might provide evidence for clinical judgement of prognosis and drug treatment.

## Data Availability Statement

Publicly available datasets were analyzed in this study. This data can be found at TCGA and GEO datasets (accession number: GSE17538).

## Author Contributions

The study’s concept and design were developed by JH and HG. CQ, WS, and HW carried out experiments and gathered data. Statistical analyses were performed and discussed by SZ, JL, DW, GL, XX, and ZS. The manuscript was written by CQ and WS. All authors contributed to the article and approved the submitted version.

## Conflict of Interest

The authors declare that the research was conducted in the absence of any commercial or financial relationships that could be construed as a potential conflict of interest.

## Publisher’s Note

All claims expressed in this article are solely those of the authors and do not necessarily represent those of their affiliated organizations, or those of the publisher, the editors and the reviewers. Any product that may be evaluated in this article, or claim that may be made by its manufacturer, is not guaranteed or endorsed by the publisher.

## Supplementary Material

Supplementary Figure S1 The flow chart of this study.

Supplementary Figure S2 Differences of immune cell infiltration and immune-related functions or pathways **(A)**, immune cell infiltration using TIMER algorithm **(B)** and TSIs **(C)** between the two molecule subtypes.

Supplementary Figure S3 The low-risk group had a better prognosis than the high-risk group in stratification analysis based on the clinicopathological parameters, such as age **(A, B)**, gender **(C, D)**, T stage **(E, F)**, lymph node status **(G, H)**, distant metastatic status **(I, J)** and tumour stage **(K, L)**.
