# Supplementary figures and images for: Identification of Molecular Subtypes and a Prognostic Signature Based on Inflammation-Related Genes in Colon Adenocarcinoma
Source: Front Immunol. 2021 Dec 23;12:769685. doi: 10.3389/fimmu.2021.769685 (PMC8733947; doi:10.3389/fimmu.2021.769685)

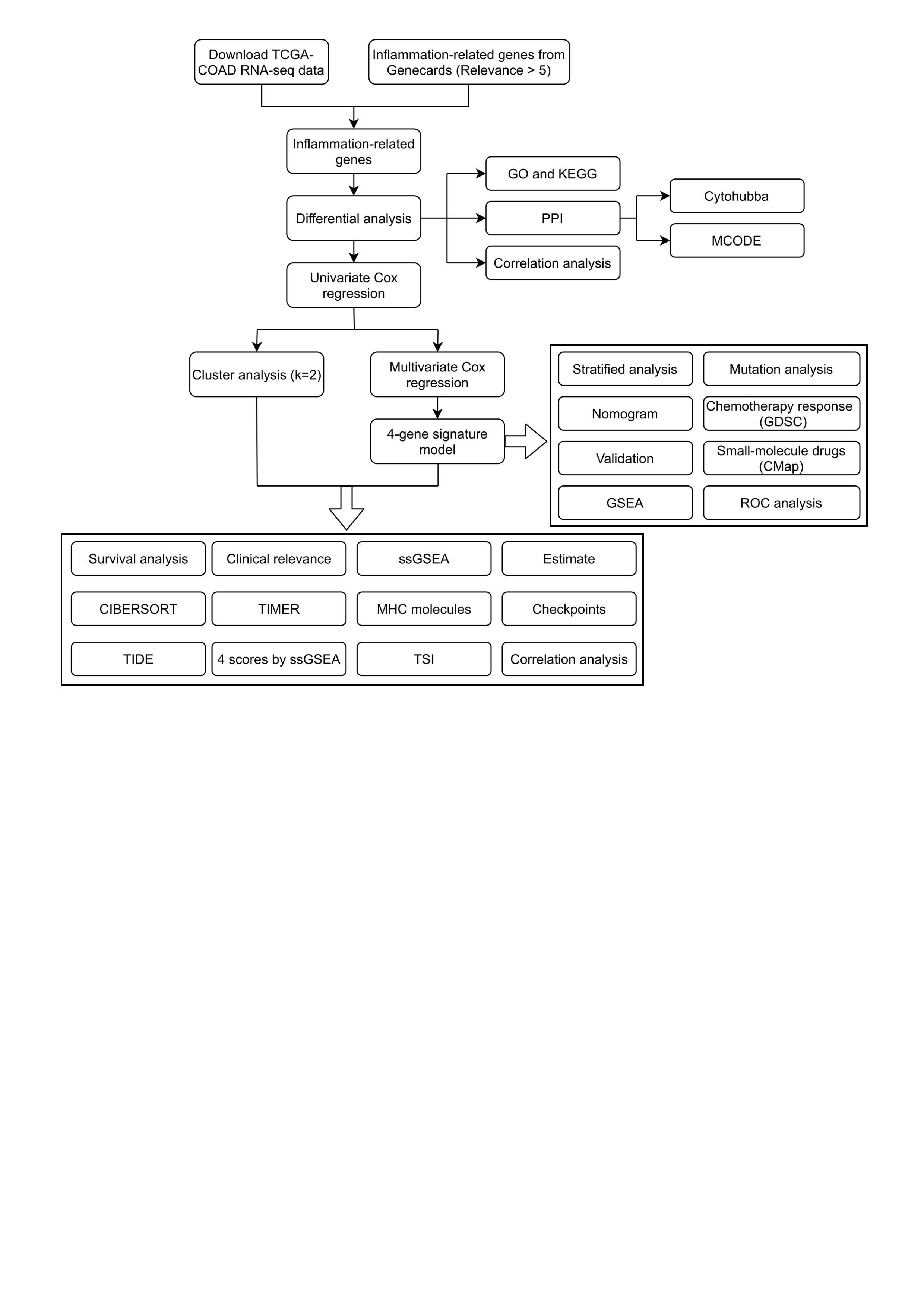

Supplement: Supplementary Figure S1 — The flow chart of this study. [file Image_1.tif]

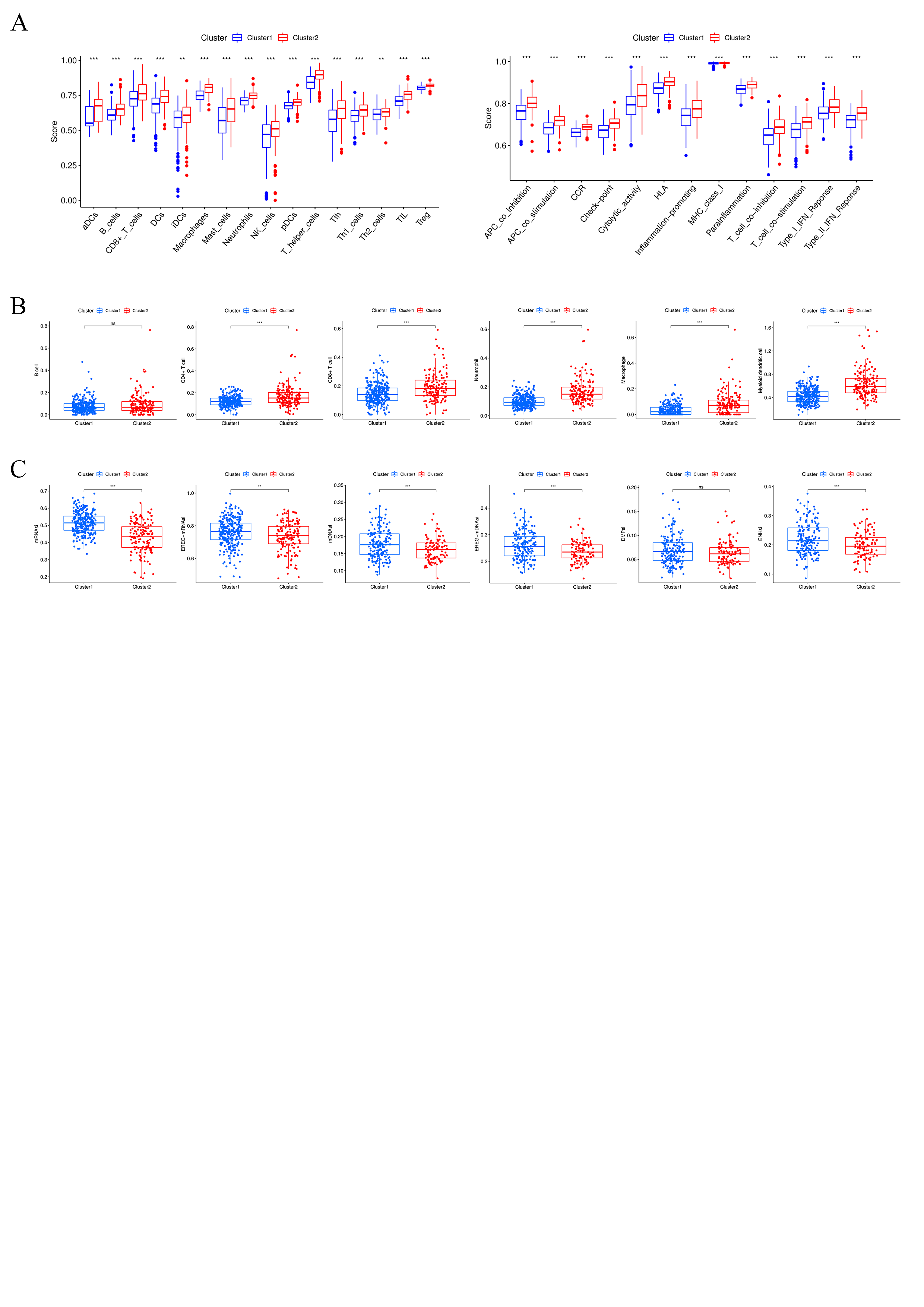

Supplement: Supplementary Figure S2 — Differences of immune cell infiltration and immune-related functions or pathways (A), immune cell infiltration using TIMER algorithm (B) and TSIs (C) between the two molecule subtypes. [file Image_2.tif]

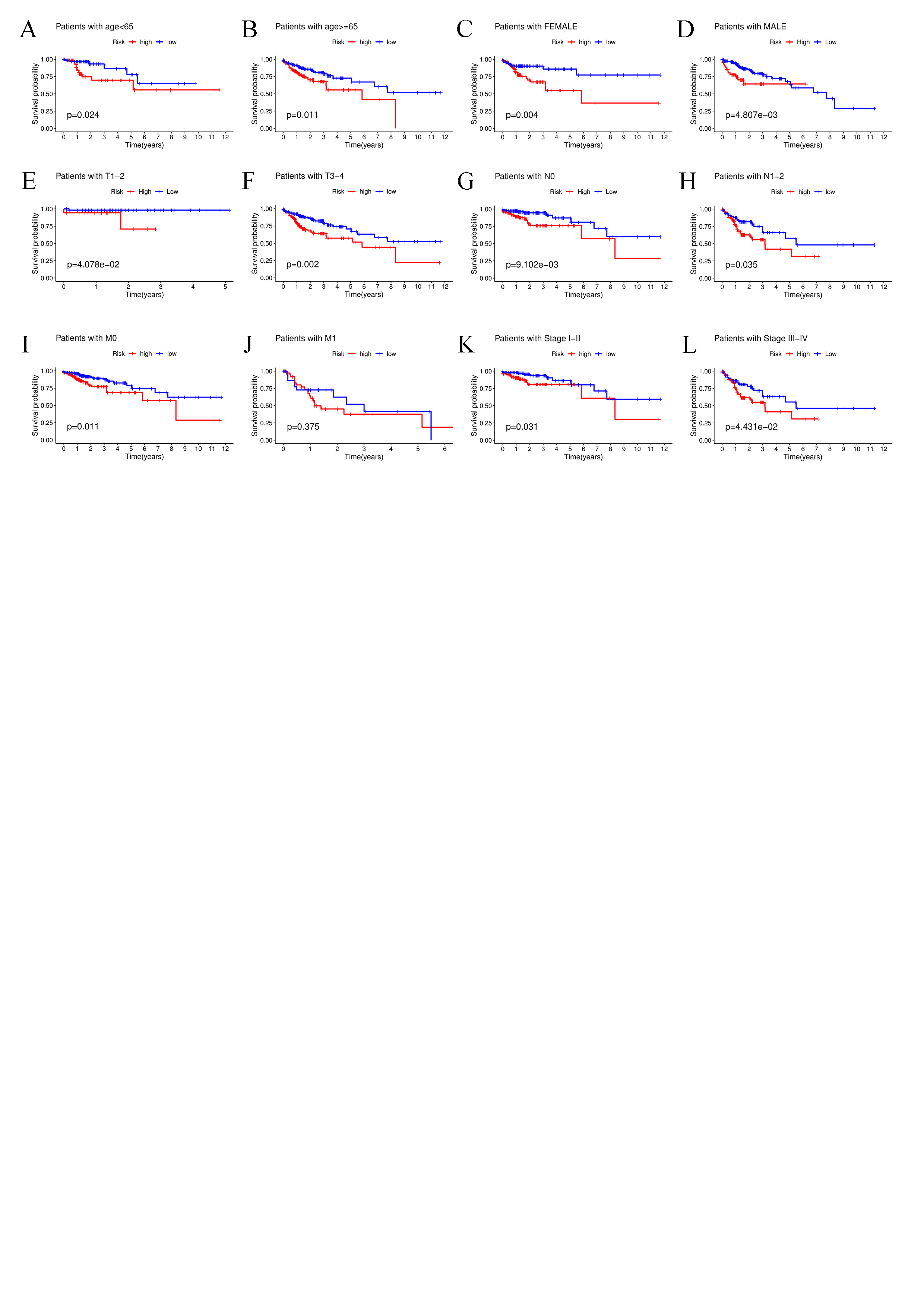

Supplement: Supplementary Figure S3 — The low-risk group had a better prognosis than the high-risk group in stratification analysis based on the clinicopathological parameters, such as age (A, B), gender (C, D), T stage (E, F), lymph node status (G, H), distant metastatic status (I, J) and tumour stage (K, L). [file Image_3.tif]
